# Ribophorin II promotes the epithelial–mesenchymal transition and aerobic glycolysis of laryngeal squamous cell carcinoma via regulating reactive oxygen species-mediated Phosphatidylinositol-3-Kinase/Protein Kinase B activation

**DOI:** 10.1080/21655979.2022.2036914

**Published:** 2022-02-14

**Authors:** Jingchun Zhou, Jingjing Zhang, Wei Zhang, Zhaoyang Ke, Yanlu Lv, Bo Zhang, Zhifang Liao

**Affiliations:** a Department of Otorhinolaryngology, Shenzhen People’s Hospital (The Second Clinical Medical College, Jinan University; The First Affiliated Hospital, Southern University of Science and Technology), Shenzhen, Guangdong, China; b Department of Otorhinolaryngology, Peking University Shenzhen Hospital, Shenzhen, China

**Keywords:** RPN2, laryngeal squamous cell carcinoma, EMT, aerobic glycolysis, ROS/PI3K/AKT

## Abstract

Ribophorin II (RPN2), a part of an N-oligosaccharyl transferase complex, plays vital roles in the development of multiple cancers. Nevertheless, its biological role in laryngeal squamous cell carcinoma (LSCC) remains unclear. The RPN2 expression levels in LSCC tissues and cell lines (AMC-HN-8 and TU212) were measured using real-time PCR, immunohistochemistry, or Western blot. The influences of RPN2 on the proliferation, migration, epithelial–mesenchymal transition, and aerobic glycolysis of LSCC cells were investigated after upregulation or downregulation of RPN2 *in vitro* and *in vivo*. Mechanically, we assessed the impact of RPN2 on the reactive oxygen species (ROS)/Phosphatidylinositol-3-Kinase (PI3K)/Protein Kinase B (Akt) signaling pathway. We found that compared with the control, RPN2 was highly expressed in LSCC tissues and cells. Overexpression of RPN2 elevated the proliferation, migration, glucose uptake, lactate production release, and levels of Vimentin, hexokinase-2 (HK-2), pyruvate dehydrogenase kinase 1 (PDK1), lactate dehydrogenase A (LDHA), and ROS, but inhibited E-cadherin expression in AMC-HN-8 cells. Knockdown of RPN2 in TU212 cells showed opposite effects on the above indexes. Meanwhile, RPN2 upregulation increased the levels of p-PI3K/PI3K and p-Akt/Akt, which were attenuated by N-acetyl-L-cysteine (NAC), an ROS inhibitor. Both NAC and PI3K inhibitor LY294002 could reverse the effects of RPN2 overexpression on the malignant phenotypes of LSCC cells. In xenografted mice, silencing RPN2 expression reduced tumor growth, ROS production, and levels of Ki-67, Vimentin, LDHA, and p-Akt/Akt, but enhanced E-cadherin expression. In conclusion, our data suggested that RPN2 promoted the proliferation, migration, EMT, and glycolysis of LSCC via modulating ROS-mediated PI3K/Akt activation.

## Graphical abstract

**Figure:**
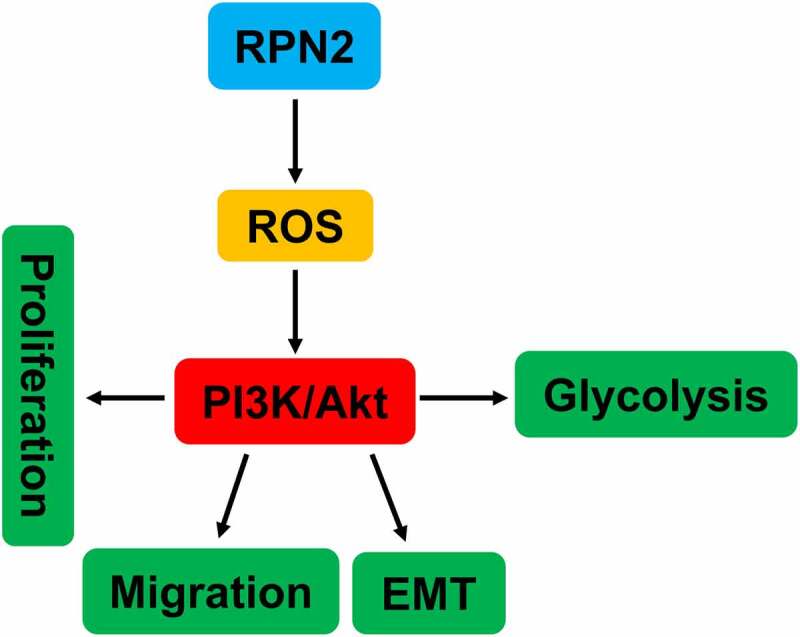

## 1 Introduction

Laryngeal squamous cell carcinoma (LSCC) is the second most common malignant tumors of the head and neck. It originates from the laryngeal mucosal epithelium and occupies about 90% of the larynx cancer, which accounts for the death of 99,840 people worldwide in 2020 [1–3]. Although the diagnostic technology has been improved, approximately 60% patients are diagnosed at advanced stages of LSCC [4]. Once the tumor has metastasized, LSCC patients will suffer from poor prognostic outcomes [4]. Hence, uncovering the underlying mechanisms of the initiation and progression of LSCC contributes to exploring diagnostic biomarkers and novel therapeutic targets for this disease.

Ribophorin II (RPN2) is a part of an N-oligosaccharyl transferase complex [5]. Studies have demonstrated that RPN2 was closely related to the development of multiple cancers [6–8]. For example, RPN2 expression was positively correlated with clinically aggressive features of breast cancer [9], and it contributed to the initiation and metastasis of breast tumor [6]. Highly expressed RPN2 was found in bladder cancers, and higher RPN2 expression was associated with patients’ tumor T stage, lymph node metastasis, and pathological degree of tumor differentiation [8]. A recent study revealed that elevated RPN2 expression was related to a poor response to induction chemotherapy in locally advanced p-16 negative head and neck squamous cell carcinoma [10]. A microarray analysis performed by Lian et al. [11] showed that RPN2 was one of the differently expressed functional genes involved in regional lymph node metastasis in LSCC. However, the exact biological role of RPN2 in LSCC was still not fully understood.

Cancer cells exhibit a unique metabolic manner to facilitate cancer progression. That is, compared with normal cells, cancer cells take up abundant glucose to produce lactate even in the circumstance of adequate oxygen, which is well known as the Warburg effect [12]. We hypothesized that RPN2 functioned in the malignant progression in LSCC. In this study, we intended to investigate the role and related mechanism of RPN2 in LSCC. The expression profiles of RPN2 in LSCC tissues and cell lines were evaluated. Using gain- and loss-of-function manipulations, the effects of RPN2 on the malignant phenotypes and glycolytic metabolism in LSCC were investigated both *in vitro* and *in vivo*. Furthermore, the molecular mechanism of RPN2 in LSCC was explored.

## 2 Materials and methods

### 2.1 Human LSCC tissues collection

A total of 25 pairs of LSCC and corresponding adjacent non-tumor tissues were obtained from patients who underwent surgery at Shenzhen People’s Hospital and Peking University Shenzhen Hospital. This study was approved by the ethical and legal standards of Shenzhen People’s Hospital (2018-0015). A part of fresh specimens was immediately frozen in liquid nitrogen for real-time PCR (qRT-PCR) analysis, and other part of tissues was fixed in 10% formalin for immunohistochemistry. None of the patients received chemotherapy or radiotherapy prior to surgery. All participants have provided written informed consent.

### 2.2 Cell culture

Two human LSCC cell lines, including AMC-HN-8 and TU212, were bought from the cell bank of the Chinese Academy of Sciences (Shanghai, China). Normal human bronchial epithelial cells (NHBEC) were purchased from ATCC (Manassas, VA, USA). These cells were grown in Dulbecco’s Modified Eagle’s medium (DMEM) containing 10% fetal bovine serum (FBS, Hyclone, Logan, Utah, USA), 1% penicillin/streptomycin in a CO_2_ incubator set to 37°C.

### 2.3 Plasmid construction and transfection

RPN2 overexpressing plasmid was constructed by subcloning the coding sequence of RPN2 into the pcDNA3.1 plasmid. RPN2 shRNA (sh-RPN2) vector was generated by cloning the annealed shRNA template DNA sequence into the pSUPER.retro.neo plasmid. The target sequence of RPN2 was as follows: RPN2#1 5’-GCCATCCATTAAGGAGGATCA-3’; RPN2#2 5’-GCGGTTGCAAGTCACCAATGT-3’. LSCC cells were transfected with RPN2 overexpressing/silencing plasmid or negative control vector by Lipofectamine 3000 (Invitrogen, Carlsbad, CA, USA) based on the manufacturer’s instructions. TU212 cells stably silencing RPN2 were selected by 800 μg/ml G418.

### 2.4 Cell counting Kit-8 (CCK-8) assay for cell viability

After transfecting with designated plasmid for 24 h, AMC-HN-8 or TU212 cells were digested and planted into 96-well plates (2000 cells/well), followed by culturing at 37°C for 0 h, 24 h, 48 h, or 72 h. Then, cells at each well were maintained with 10 μl of CCK-8 solution (Beyotime, Shanghai, China) for 1 h. Lastly, a microplate reader (Bio-Tek, Winooski, USA) was used to examine the absorbance at 450 nm.

### 2.5 Transwell assay

Transwell chambers (8 μm in pore size, Millipore, Burlington, USA) were used to analyze the migration of LSCC cells. Briefly, the transfected AMC-HN-8 or TU212 cells were first digested by trypsinase to obtain a single-cell suspension, and adjusted the density to 1 × 10^5^ cells/ml with FBS-free medium. Subsequently, 200 μl of cell suspension was seeded into the upper chamber, while the lower chamber was supplemented with 500 μl DMEM medium containing 20% FBS. Cells were incubated at 37°C for 24 h. After wiping cells in the upper chamber using cotton swabs and fixing with 4% paraformaldehyde, cells in the lower chamber were stained by crystal violet for 10 min. The staining cells were then photographed by a light microscope. Each chamber was randomly selected for five fields to count migrated cell numbers.

### 2.6 Reactive oxygen species (ROS) analysis

Intracellular ROS levels were analyzed by dichlorodihydrofluorescein diacetate (DCFH-DA) staining, as previously described [13]. After the indicated treatment, AMC-HN-8 or TU212 cells were incubated with 10 μmol/l DCFH-DA for 20 min at 37°C in the dark. A fluorescence microscope (Nikon, Tokyo, Japan) was used to obtain the images. The fluorescence intensity was measured using ImageJ Software.

### 2.7 qRT-PCR

TRIzol® reagent (Invitrogen) was used to extract total RNAs from tissues and cells in this study. Then, the concentration and purity of total RNAs were detected by Nanodrop 2000. cDNAs were synthesized at the base of RNAs using the reverse transcription kit (Takara, Dalian, China). Afterward, we performed qRT-PCR assay using the SYBR Premix Ex Taq II (Takara) on an Applied Biosystems 7500 Sequence Detection System (Applied Biosystems, Foster City, CA, USA). The primer sequences used are shown in Table 1. The result was calculated by the 2^−ΔΔCT^ method, with GAPDH as the internal reference.

**Table 1.** Primer sequences

Gene	Forward primer (5’-3’)	Reverse primer (5’-3’)
RPN2	CTGGGACATGCTGCTATGCT	TGCTGTTCTCTTGACTGCCT
HK-2	GAGCCACCACTCACCCTACT	CCAGGCATTCGGCAATGTG
LDHA	TTGACCTACGTGGCTTGGAAG	GGTAACGGAATCGGGCTGAAT
PDK1	GAGAGCCACTATGGAACACCA	GGAGGTCTCAACACGAGGT
GAPDH	CTGGGCTACACTGAGCACC	AAGTGGTCGTTGAGGGCAATG

### 2.8 Xenograft animal studies

All animal studies were approved by the Institutional Animal Care and Use Committee of Shenzhen People’s Hospital (2018-0015). Four- to six-week-old male BALB/C nude mice were purchased from Guangdong Medical Laboratory Animal Center. All animals were maintained in a specific pathogen-free facility and allowed free access to food and water. After a week of acclimatization, the mice were divided into sh-RPN2 group and sh-NC group (n = 5 per group) randomly. 1 × 10^7^ TU212 cells (200 μl) stably transfecting sh-RPN2 or sh-NC were subcutaneously injected into the right flank of nude mice. Seven days later, the length and width of the tumors were measured using a vernier caliper every 4 days, and tumor volumes were calculated by the formula: length × width^2^ × 0.5. The mice were euthanized on day 23 by cervical dislocation under anesthesia with isoflurane. Tumor tissues were dissected for further analysis.

### 2.9 Immunohistochemical (IHC) staining

Tumor samples were fixed with 4% paraformaldehyde, embedded in paraffin, and cut into 5 µm thick slices. Samples were rehydrated for IHC according to a standard protocol as described previously [14]. After antigen retrieval, sections were stained with primary antibodies, including RPN2 (Cat. No. ab244399, 1:200, Abcam, Cambridge, MA, USA), Ki-67 (Cat. No. ab16667, 1:300, Abcam), E-cadherin (Cat. No. ab1416, 1:200, Abcam), Vimentin (Cat. No. #5741, 1:3000, Cell Signaling Technology, Danvers, MA, USA), and LDHA (Cat. No. #3582, 1:500, Cell Signaling Technology) overnight at 4°C. Then, sections were stained with secondary antibodies and 3,3’-diaminobenzidine. After counterstained with hematoxylin, the slices were imaged using a light microscope. The staining intensity was scored by the semi-quantitative H-score by two independent observers [14].

### 2.10 Western blot

Proteins were extracted from cells and tissues using a RIPA lysis buffer containing protease inhibitor. Total proteins (30 µg from each sample) were subjected to 10% sodium dodecyl sulfate polyacrylamide gel electrophoresis, which could separate proteins according to the molecular weight, after determining the concentrations using a BCA protein assay kit (Beyotime). Proteins in the gel were transferred to polyvinylidene difluoride membranes. Subsequently, the membranes were blocked with 5% nonfat milk for 1 h and incubated with primary antibodies against RPN2 (1:800, Abcam), E-cadherin (1:1000, Abcam), Vimentin (1:1000, Cell Signaling Technology), Phosphatidylinositol-3-Kinase (PI3K; Cat. No. #4255, 1:1000, Cell Signaling Technology), p-PI3K (Cat. No. #17366, 1:1000, Cell Signaling Technology), Protein Kinase B (Akt; Cat. No. #4691, 1:1000, Cell Signaling Technology), p-Akt (Cat. No. #4060, 1:2000, Cell Signaling Technology), and GAPDH (Cat. No. ab181603, 1:10,000, Abcam) overnight at 4°C. Next, horseradish peroxidase (HRP)-labeled secondary antibodies were added to the membranes for further incubation. One hour later, the protein bands were developed by electrochemical luminescence kit (Beyotime) and observed using a Gel Doc 2000 (Berkeley, California, USA).

### 2.11 Statistical analysis

The results are presented as the mean ± standard deviation (SD). SPSS 20.0 was used to analyze all statistical analyses. The expression of RPN2 in paired LSCC tumor and adjacent non-tumor samples was assayed by the nonparametric paired Wilcoxon t-test. Data among the groups were determined by Student’s t-test or one-way analysis of variance. Differences with *P* < 0.05 were regarded as significant.

## 3 Results

### 3.1 RPN2 was highly expressed in LSCC tissues and cells

**Figure 1.**
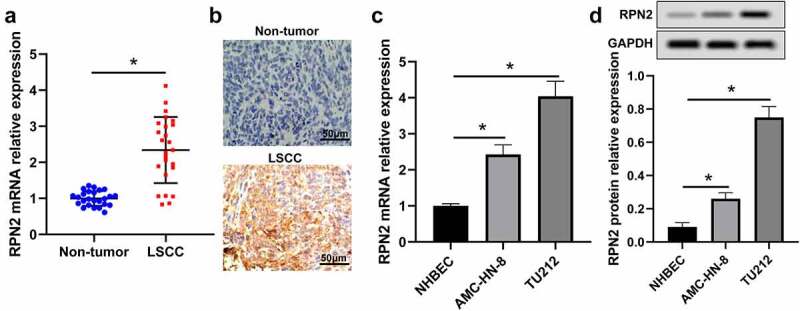
RPN2 expression was augmented in human LSCC tissues and cells. (a) qRT-PCR assay for RPN2 mRNA expression in 25 paired LSCC tissues and adjacent non-tumor samples. (b) IHC analysis for the protein level of RPN2 in collected human samples. RPN2 mRNA (c) and protein (d) levels were detected in LSCC cell lines (TU212 and AMC-HN-8) and normal human bronchial epithelial NHBEC cells. *P < 0.05.

To explore the role of RPN2 in LSCC, RPN2 expression levels in LSCC tissues and cells were measured. RPN2 mRNA expression in 25 LSCC tissues was higher than that in adjacent non-tumor specimens, which was detected by qRT-PCR (Figure 1(a)). RPN2 expression was also confirmed by the IHC method in these samples (Figure 1(b)). Additionally, we found that the expression of RPN2 was significantly increased in LSCC cell lines (TU212 and AMC-HN-8) when compared to NHBEC cells (Figure 1(c,d)).

### 3.2 RPN2 promoted the proliferation, migration, and epithelial–mesenchymal transition (EMT) of LSCC cells

**Figure 2.**
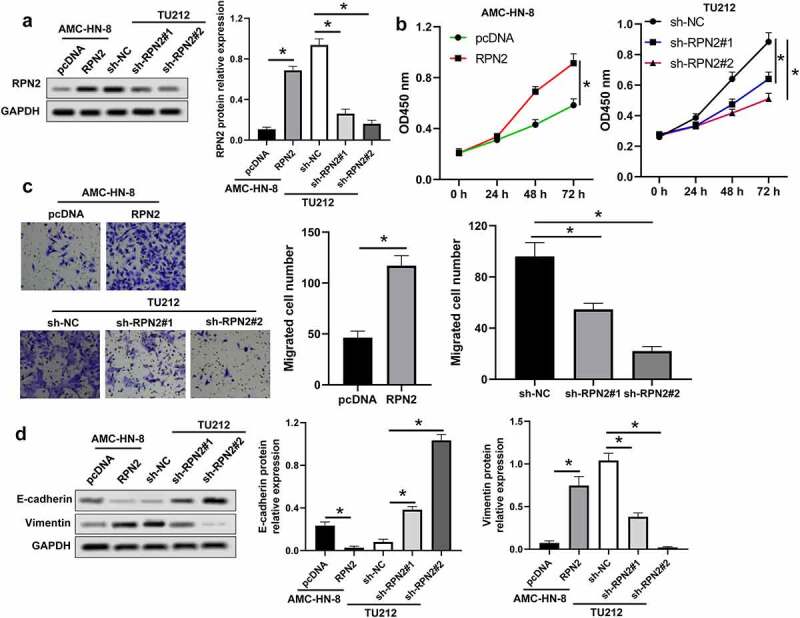
RPN2 facilitated the tumorigenesis of LSCC cells. (a) Western blot assay for RPN2 protein levels in LSCC cells after overexpressing or silencing RPN2. (b) The viabilities of AMC-HN-8 cells transfected with RPN2 overexpressing or control pcDNA plasmid and TU212 cells transfected with RPN2 silencing or control sh-NC vector were analyzed by CCK-8 assay. (c) Cell migration was determined by Transwell analysis. (d) Protein levels of E-cadherin and Vimentin in LSCC cells were investigated by Western blot. *P < 0.05.

The biological effects of RPN2 on LSCC were assessed in LSCC cells transfected with pcDNA3.1-RPN2 or sh-RPN2 plasmid. pcDNA3.1-RPN2 plasmid transfection significantly elevated RPN2 expression, while sh-RPN2 decreased the expression of RPN2 (Figure 2(a)). Upregulation of RPN2 led to increases in the viability and migration of AMC-HN-8 cells (Figure 2(b,c)). Contrarily, downregulation of RPN2 caused suppression of TU212 cell viability and migration (Figure 2(b,c)).

The involvement of RPN2 in the EMT of LSCC cells was analyzed, as indicated in Figure 2(d), and RPN2 upregulation dramatically inhibited E-cadherin expression, but elevated Vimentin expression in AMC-HN-8 cells. Knockdown of RPN2 caused an increase of E-cadherin, and a decrease of Vimentin in TU212 cells (Figure 2(d)). These results demonstrated that RPN2 could induce the proliferation, migration, and EMT of LSCC cells.

### 3.3 Downregulation of RPN2 attenuated the glycolytic process of LSCC cells

**Figure 3.**
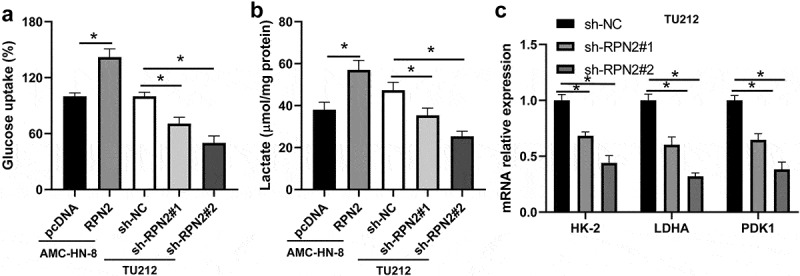
RPN2 contributed to the glycolysis of LSCC cells. (a) LSCC cells were transfected with RPN2 overexpressing or silencing plasmid, and glucose uptake was analyzed. (b) The release of lactate production was measured in AMC-HN-8 cells and TU212 cells. (c) Downregulation of RPN2 in TU212 cells inhibited the mRNA expression of HK-2, LDHA, and PDK1, which was examined by qRT-PCR. *P < 0.05.

Glycolysis is closely connected with the proliferation and metastasis of tumor cells [15]. Therefore, this study assessed the effect of RPN2 on the glycolytic process of LSCC cells. Upregulation of RPN2 significantly elevated glucose uptake rate and the release of lactate in AMC-HN-8 cells (Figure 3(a,b)). The downregulation of RPN2 caused the suppression of glucose uptake and lactate release in TU212 cells (Figure 3(a,b)). Consistently, levels of many key glycolytic enzymes, such as hexokinase-2 (HK-2), pyruvate dehydrogenase kinase 1 (PDK1), and lactate dehydrogenase A (LDHA), were inhibited after RPN2 downregulation in LSCC cells (Figure 3(c)). These data indicated that silencing RPN2 expression attenuated glycolysis of LSCC cells.

### 3.4 RPN2 regulated the migration, EMT, and glycolysis of LSCC cells via modulating ROS production

**Figure 4.**
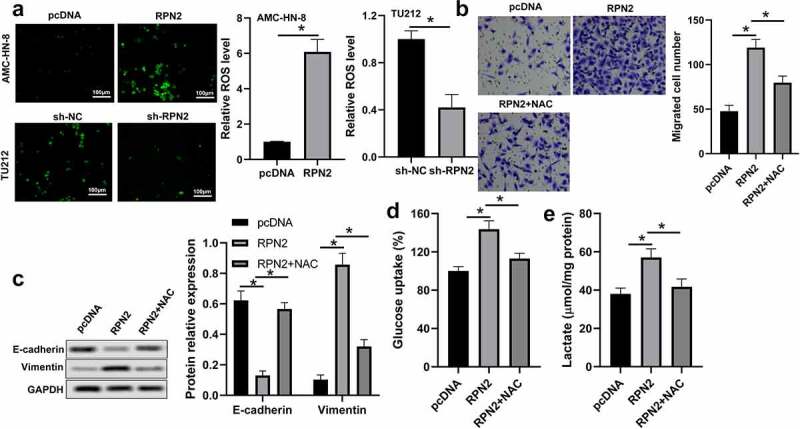
RPN2 functioned via modulating ROS level in LSCC cells. (a) Intracellular ROS levels were examined by DCFH-DA fluorescence. (b) AMC-HN-8 cells transfected with RPN2 overexpressing plasmid were treated with 10 mmol/l NAC for 24 h. Cell migration was assayed by Transwell analysis. (c) Protein levels of E-cadherin and Vimentin were assayed by Western blot in AMC-HN-8 cells after different treatments. (d) Glucose uptake was measured in AMC-HN-8 cells. (e) Lactate production release was evaluated in AMC-HN-8 cells. *P < 0.05.

Studies have revealed that ROS within a specific range could facilitate tumor development and progression by regulating proliferation, metastasis, and glycolysis [16,17]. Therefore, we analyzed the influence of RPN2 on ROS level. As shown in Figure 4(a), upregulation of RPN2 is elevated, whereas downregulation of RPN2 reduced ROS levels in LSCC cells. An ROS inhibitor N-acetyl-L-cysteine (NAC) was used to further verify the role of ROS in RPN2 functioning. The results of Figure 4(b–e) demonstrated that NAC treatment significantly prevented RPN2-promoted migration, EMT, and glycolysis in LSCC cells.

### 3.5 RPN2 functioned via activating the PI3K/Akt pathway through increasing ROS

**Figure 5.**
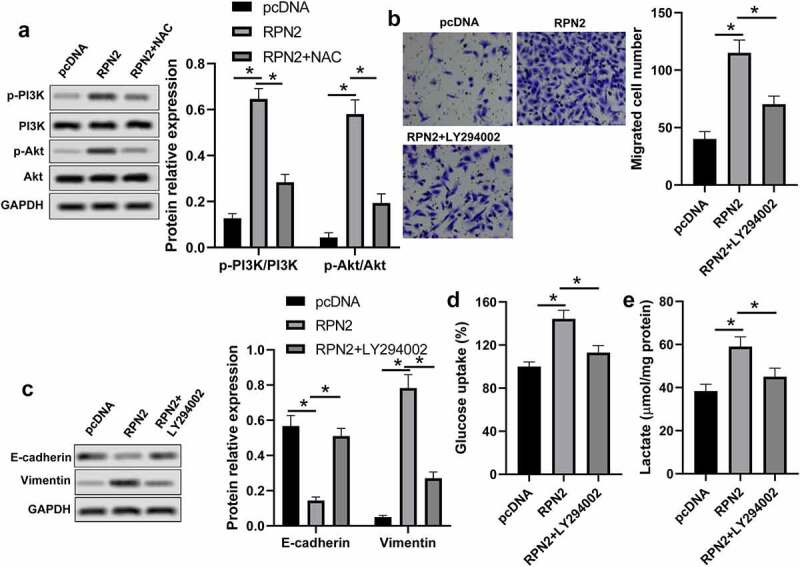
RPN2 regulated the characteristics of LSCC via activating the PI3K/Akt pathway through elevating ROS. (a) Western blot was used to measure the protein levels of p-PI3K, PI3K, p-Akt, and Akt. (b) AMC-HN-8 cells that transfected with RPN2 overexpressing or pcDNA control plasmid for 48 h were treated with or without LY294002 (2 μmol/l) for 24 h, and the migration of these cells was analyzed by Transwell assay. (c) EMT-related proteins E-cadherin and Vimentin were measured by Western blot. Glucose uptake (d) and lactate release (e) were determined by appreciate kit, respectively. *P < 0.05.

PI3K/Akt signaling is a vital pathway in cancer cells that regulates cell growth, metastasis, and glycolysis [18,19]. The ROS is associated with the activation of PI3K/Akt signaling [20]. Therefore, we further analyzed the impact of RPN2 on PI3K/Akt pathway activation. As demonstrated in Figure 5(a), the levels of p-PI3K/PI3K and p-Akt/Akt were elevated in LSCC cells with RPN2 overexpressing, the levels of which were significantly suppressed by NAC. A PI3K inhibitor LY294002 was used to confirm whether RPN2 functioned via modulating the PI3K/Akt pathway. The results revealed that LY294002 dramatically recovered the impacts of RPN2 upregulation on the migration, EMT, and glycolysis in LSCC cells (Figure 5(b–e)). Taken together, these data suggested that RPN2 played roles in LSCC via activating the PI3K/Akt pathway through elevating ROS level.

### 3.6 Suppression of RPN2 inhibited the tumorigenicity of LSCC cells in vivo

**Figure 6.**
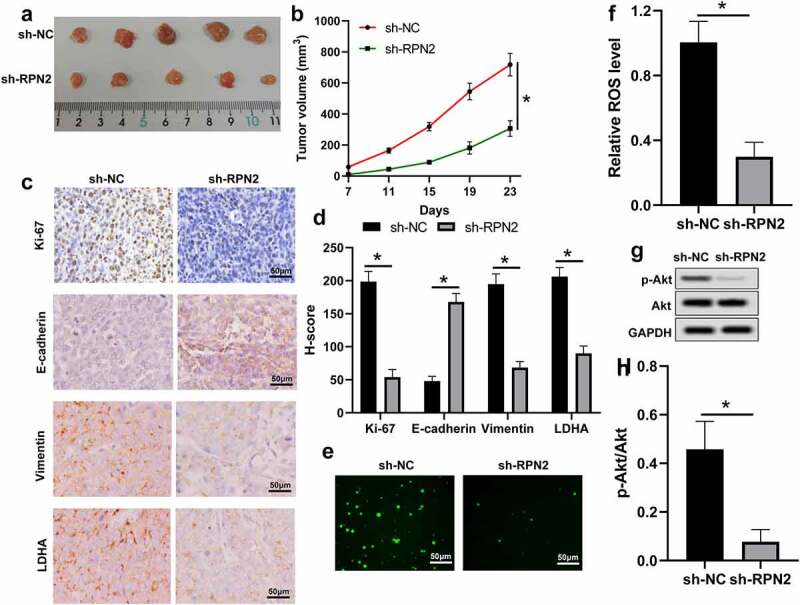
Knockdown of RPN2 suppressed the tumorigenicity of LSCC cells in a xenograft tumor model. (a) Mice were sacrificed on day 23 and tumor images were exhibited. (b) Tumor volume of each mouse was calculated every 4 days from 7 days after implantation. (c) IHC assay for the levels of Ki-67, E-cadherin, Vimentin, and LDHA in mouse tumor tissues, and (d) H-score was used to measure the staining intensity. (e and f) ROS levels in mouse tumor samples were analyzed. (g and h) p-Akt/Akt level was downregulated by sh-RPN2 in tumors. *P < 0.05.

The influence and mechanism of RPN2 in LSCC were further verified in an established xenograft tumor model using TU212 cells stably silencing RPN2 or control. Tumors with RPN2 knockdown showed smaller volumes than those with sh-NC (Figure 6(a,b)). Through the IHC analysis, the results revealed that levels of Ki-67, Vimentin, and LDHA were suppressed, whereas E-cadherin expression was enhanced in mouse tumors after RPN2 knockdown (Figure 6(c,d)). Additionally, the ROS level and the activation of Akt were both inhibited by sh-RPN2 (Figure 6(e–h)). These data indicated that downregulation of RPN2 could inhibit the tumorigenicity of LSCC cells *in vivo*.

## 4 Discussion

In the current study, we found that RPN2 was highly expressed in LSCC tissues than that in adjacent non-tumor samples. In addition, compared with the NHBEC cells, there was an increased tendency of RPN2 expression in TU212 and AMC-HN-8 LSCC cell lines. Overexpression of RPN2 promoted the proliferation, migration, EMT, and glycolysis of LSCC cells. Contrarily, downregulation of RPN2 suppressed these characteristics of LSCC cells. Our findings revealed that RPN2 functioned via ROS-mediated activation of the PI3K/Akt pathway.

Human RPN2 gene is located on chromosome 20q12-q13.1. It is one of the highly conserved integral rough endoplasmic reticulum-specific membrane glycoproteins. RPN2 is responsible for the translocation of secretory proteins and the specificity of endoplasmic reticulum [21]. Accumulating studies have verified that RPN2 acted as an oncogene in many types of cancers, for example, colon cancer [22], breast cancer [23], esophageal cancer [24], and nasopharyngeal carcinoma [25]. This study showed that RPN2 was overexpressed in LSCC tissues and cells. Given that Chen et al. [10] found that p-16 negative locally advanced head and neck squamous cell carcinoma patients with RPN2 overexpression exhibited poor responses to induction chemotherapy, and revealed a shorter survival, we hypothesized that RPN2 might be a carcinogenic gene in LSCC. Our results showed that the increase in RPN2 significantly promoted the proliferation and migration of LSCC cells. While the knockdown of RPN2 not only inhibited the proliferation and migration of LSCC cells *in vitro* but also suppressed tumor growth and the expression of Ki-67, a tumor proliferation marker that was associated with tumoral aggressiveness and poor prognosis in LSCC patients [26], in a xenograft tumor model. During EMT, carcinoma cells lose their polarized epithelial features and transform into mesenchymal phenotypes to acquire malignant properties, including metastasis, cancer stem cell activity, and therapy resistance [27]. Hence, we further analyzed the involvement of RPN2 in the EMT of LSCC cells by evaluating levels of two EMT-related genes (E-cadherin and Vimentin). The results indicated that upregulation of RPN2 decreased E-cadherin expression but augmented Vimentin level in LSCC cells. On the contrary, knockdown of RPN2 notably enhanced E-cadherin expression, whereas suppressed Vimentin expression both in LSCC cells and in mouse tumors. These data suggested that RPN2 was an oncogene in LSCC.

Aerobic glycolysis reflects the malignant progression of a majority of tumors, including LSCC [28]. HK-2, one of the most important glycolytic enzymes in the first rate-limiting step of glucose metabolism that mediate the phosphorylation of glucose to glucose-6-phosphate, is over-expressed in multiple tumors [29]. PDK1 is a critical glycolysis enzyme that modulates the switch of glucose oxidation to glycolysis [30]. It was upregulated in LSCC tissues, and elevated PDK1 expression was associated with the progression of LSCC [31]. LDHA irreversibly catalyzes the conversion of pyruvate to lactate to promote the glycolysis process [32]. We found that RPN2 upregulation significantly increased, whereas its downregulation suppressed, glucose uptake rate and lactate release in LSCC cells. Silencing of RPN2 also inhibited the expression of HK-2, PDK1, and LDHA in these cells and decreased LDHA level in mouse tumor tissues. In general, these results demonstrated that RPN2 could promote the glycolysis process in LSCC.

Although ROS overabundance limits tumor growth, maintaining ROS within a narrow range can facilitate the tumorigenic characteristics of cancer cells [16,17]. This study found that ROS level was promoted by RPN2 overexpression but reduced after RPN2 downregulation in LSCC cells and/or mouse models. Moreover, an ROS inhibitor NAC abrogated the impacts of RPN2 upregulation on the migration, EMT, and glycolysis in LSCC cells. Constitutive activation of the PI3K/Akt pathway is often seen in multiple human cancers, which plays an essential role in maintaining tumor cell growth, metastasis, EMT, and metabolism [18,19,33]. This pathway can be activated by the increase in ROS. Han et al. [8] found that RPN2 promoted the growth and metastasis of bladder cancer by activating the PI3K/Akt pathway. Hence, the influence of RPN2 on the PI3K/Akt pathway in LSCC was further explored. As expected, overexpression of RPN2 dramatically elevated the levels of p-PI3K/PI3K and p-Akt/Akt in LSCC cells, and suppression of RPN2 decreased the ratio of p-Akt to Akt both *in vitro* and *in vivo*. Moreover, the PI3K inhibitor LY294002 recovered the influences of RPN2 upregulation on the migration, EMT, and glycolysis in LSCC cells. Interestingly, ROS inhibitor NAC significantly attenuated the activation of PI3K/Akt pathway affected by RPN2. In general, these results suggested that RPN2 functioned via ROS-mediated activation of the PI3K/Akt pathway.

## 5 Conclusions

Our results demonstrated that RPN2 was overexpressed in LSCC, and it could promote the malignant phenotypes and glycolysis of LSCC cells *in vitro* and *in vivo* via activating the PI3K/Akt pathway through increasing ROS level. Chemoresistance is another main factor resulting in the poor prognosis of LSCC patients [34], and RPN2 has been verified to be related to chemoresistance in many cancers [23,35,36]; hence, it is of great value to further investigate the relationship between RPN2 and chemoresistance in LSCC. Altogether, targeting RPN2 may serve as a potential therapy for LSCC.

## Highlights

1. RPN2 was highly expressed in LSCC tissues and cells.

2. RPN2 promoted the progression of LSCC both *in vitro* and *in vivo.*

3. Downregulation of RPN2 attenuated the glycolytic process of LSCC cells.

4. RPN2 regulated the malignant phenotypes of LSCC via modulating ROS production.

5. RPN2 activated the PI3K/Akt pathway through increasing ROS.
